# Cessation and resumption of elective neurointerventional procedures during the coronavirus disease 2019 pandemic and future pandemics

**DOI:** 10.1177/15910199211035900

**Published:** 2021-11-08

**Authors:** Tim W Malisch, Sameer A Ansari, Gary R Duckwiler, Kyle M Fargen, Steven W Hetts, Franklin A Marden, Athos Patsalides, Clemens M Schirmer, Allan Brook, Justin F Fraser

**Affiliations:** 1 21863 AMITA Alexian Brothers Medical Center, USA; 2 Departments of Radiology, Neurosurgery, and Neurology, Feinberg School of Medicine, 12244 Northwestern University, USA; 3 Interventional Neuroradiology, David Geffen School of Medicine, University of California in Los Angeles, USA; 4 Departments of Neurological Surgery and Radiology, 8676 Wake Forest University, USA; 5 Departments of Radiology and Biomedical Engineering, University of California in San Francisco, USA; 6 Department of Neurosurgery, Northshore University Hospital, USA; 7 Department of Neurosurgery and Neuroscience Institute, 2780 Geisinger Health System and Geisinger Commonwealth School of Medicine, USA; 8 Research Institute of Neurointervention, Paracelsus Medical University, Austria; 9 Departments of Radiology and Neurosurgery, 2013 Albert Einstein College of Medicine and Montefiore Medical Center, USA; 10 Departments of Neurological Surgery, Neurology, Radiology, and Neuroscience, University of Kentucky, USA

**Keywords:** COVID-19, neurointervention, clinical service

## Abstract

At the time of this writing, the coronavirus disease 2019 pandemic continues to be a global threat, disrupting usual processes, and protocols for delivering health care around the globe. There have been significant regional and national differences in the scope and timing of these disruptions. Many hospitals were forced to temporarily halt elective neurointerventional procedures with the first wave of the pandemic in the spring of 2020, in order to prioritize allocation of resources for acutely ill patients and also to minimize coronavirus disease 2019 transmission risks to non-acute patients, their families, and health care workers. This temporary moratorium on elective neurointerventional procedures is generally credited with helping to “flatten the curve” and direct scarce resources to more acutely ill patients; however, there have been reports of some delaying seeking medical care when it was in fact urgent, and other reports of patients having elective treatment delayed with the result of morbidity and mortality. Many regions have resumed elective neurointerventional procedures, only to now watch coronavirus disease 2019 positivity rates again climbing as winter of 2020 approaches. A new wave is now forecast which may have larger volumes of hospitalized coronavirus disease 2019 patients than the earlier wave(s) and may also coincide with a wave of patients hospitalized with seasonal influenza. This paper discusses relevant and practical elements of cessation and safe resumption of nonemergent neurointerventional services in the setting of a pandemic.

## Background

The coronavirus disease 2019 (COVID-19) virus was identified in the human population in late 2019 and rapidly spread across the globe. Although the case fatality rate of the virus was unclear, the virus was recognized to be highly transmissible. By March of 2019, many parts of the world saw their hospitals and systems of health care strained. Resources of intensive care unit (ICU) beds, ventilators, drugs (especially those used with ventilated patients), and trained staff were in short supply for the most acutely ill patients. Personal protective equipment (PPE) necessary to protect non-COVID patients, health care providers, and family members was also in short supply. Many regions of the globe adopted protocols created by governmental public health entities and major physician organizations to cease all elective, non-urgent procedures and clinic visits.^1–3^ Many such protocols included examples of hierarchies for defining elective, urgent, and emergent procedures; however, the examples did not translate into clear guidance on the diverse array of neurointerventional procedures.^4^

In the late spring and early summer of 2020 reports surfaced of morbidity and mortality rising in the non-hospitalized, non-COVID population, theorized to represent patients succumbing to urgent medical conditions due either to avoidance of seeking medical care or a delay in scheduled medical care.^5,6^ Reports also suggested a corresponding decrease in patients presenting to emergency departments with symptoms of medical emergencies including neurovascular emergencies such as acute stroke.^7^ Neurointerventional practices saw their volumes of patients presenting within the time window for stroke thrombectomy drop, despite a predicted increase in hypercoagulable complications in the COVID-19 population.^8–14^ Ambulance services and law enforcement reports suggested an increase in calls for at-home medical deaths.^7^ These reports suggest that avoidance of medical facilities is not without risk, elective medical care, and surgical procedures cannot be postponed indefinitely, and eventually many non-urgent conditions progress to urgent ones requiring treatment.

As reports across the globe now indicate COVID-19 diagnoses are again climbing,^15^ we are faced with a new wave of COVID-19 this winter, which may be exacerbated if coinciding with seasonal influenza, again straining supplies of ICU beds, ventilators, resources, personnel, and PPE.^16^ There is hope that the fatality rate of COVID-19 may be lower with this wave due to new therapeutics, a better understanding of established drugs such as steroids, delivery of supplemental oxygen without premature utilization or overutilization of ventilators, and perhaps a shift in the COVID-19 population to younger patients with fewer risk factors for an adverse outcome from the disease. But the evidence does not suggest fewer hospitalizations with this new wave of COVID-19.^17^ Thoughtful action plans are required to better address these issues. This document provides guidance during the COVID-19 pandemic, and future pandemics, on the cessation and resumption of elective neurointerventional procedures that allocates scarce resources and minimizes the risk of transmission of infection to patients, families, and health care providers while enabling health care organizations to provide optimal care to all the patients they serve. Although this pandemic of COVID-19 will at some point pass, there is a clear need to define prioritization of procedures and establishment of safe neurointerventional workflows whenever regional and institutional resources are strained.

## Cessation and resumption of elective cases: Timing at the local level

**Figure 1.**
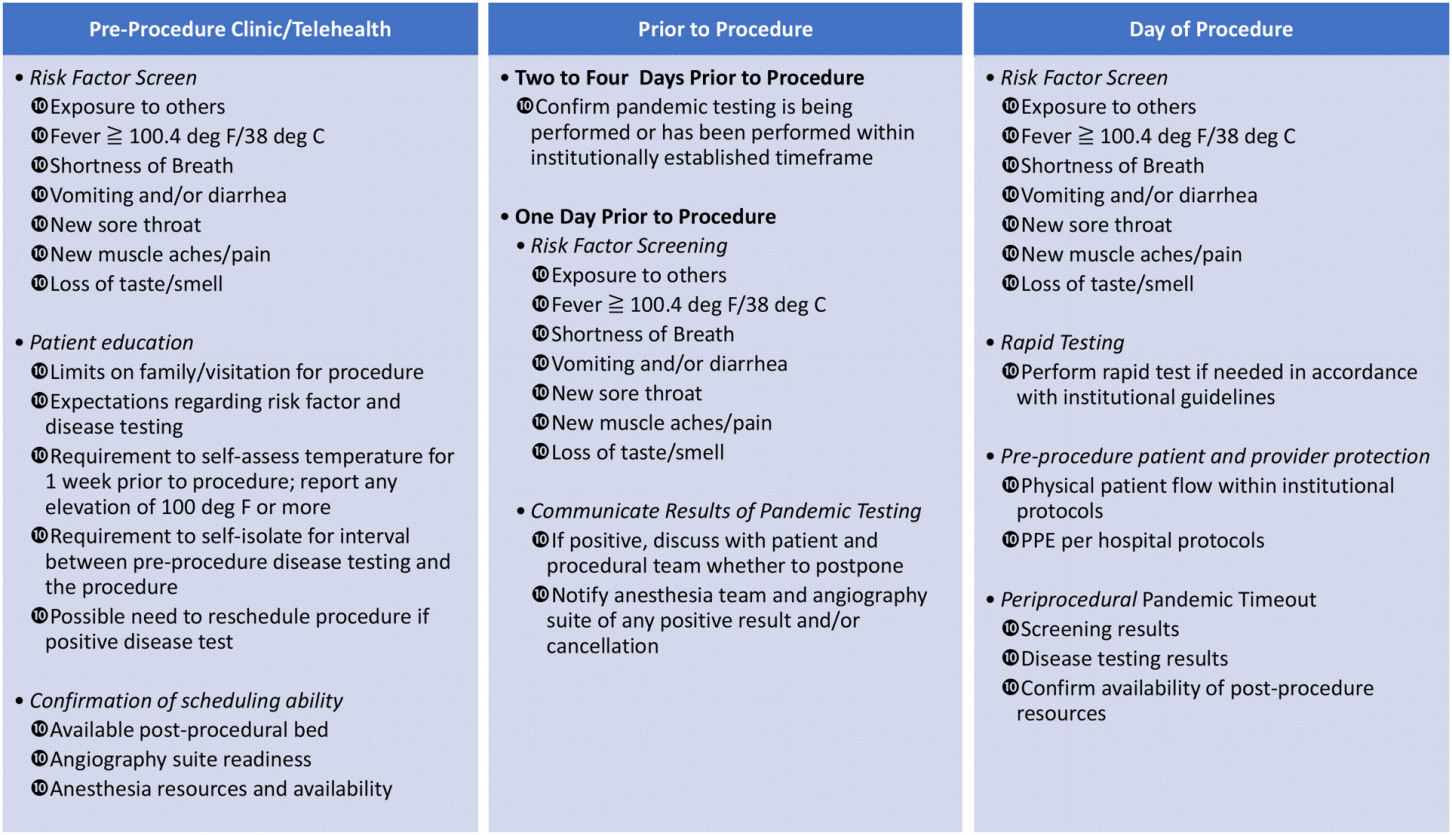
Checklist for elective neurointerventional radiology (NIR) procedures during pandemic.

When considering whether the local health system or institution can justify performance of elective, non-urgent neurointerventional procedures, institutions should follow local, regional, state, and if applicable national regulations, and should consider these local factors:

1. *Pre-hospital resources and covid-19 screening*: Providing elective procedures during a pandemic must minimize the risk of infecting those not infected—including other patients, health providers and visitors.^18–20^ Screening patients and visitors in advance of their hospital visit with risk factor screens (Figure 1) and taking temperatures may be adequate for clinic visits and procedures defined not to be aerosol generating procedures. Patients undergoing aerosol generating procedures should be required to undergo COVID-19 testing. With the global variation in resources for COVID-19 testing, the type of test required and the appropriate time window of the test relative to the date of the procedure would likely be defined according to local availability.^21–23^ After a patient is screened or tested, social distancing, and mask wearing are recommended until the time of the procedure.
2. *In-hospital resources—Sufficient for a surge in acutely ill patients*: The medical center must maintain adequate resources to safely and effectively diagnose and treat its current population of COVID-19 positive patients and persons under investigation (PUI), as well as anticipate an incremental volume of new cases without risking shortage of supplies, beds, other hospital space, and personnel.^24–27^ Fortunately, most elective neurointerventional procedure patients require shorter lengths of stay and lower intensity of care than the average severe COVID-19 patient.
3. *In-hospital resources—Sufficient to protect against transmission*: Hospital spaces to be shared by COVID-19 positive, PUI patients and COVID-19 negative patients require resource and time-consuming cleaning between patients. Workflows that minimize cross-flow of COVID-19 positive and negative patients will be more efficient but may not be feasible in smaller institutions. When community COVID-19 levels warrant concern about asymptomatic spreaders, then in addition to screening visitors as discussed above, public areas within the hospital available to visitors should have sufficient space to allow adequate social distancing. Without the latter, performance of elective procedures may need to be performed without allowing visitors. This may also apply to clinic visits.

## Prioritization of non-emergent procedures

It is recommended that both individual physician practices and hospitals adopt a system of prioritization for performance of procedures, taking into account both the scarcity of resources required for the procedure and the risk to the patient in delaying the procedure. Such a protocol should be transparent across clinical service lines and focused on a comparative patient risk assessment. This facilitates an open discussion among services that may need to share resources. Several such categorization schemes have been published which may serve as a foundation for such discussions.^27–30^ We propose a tiered system that includes examples specific to neurointerventional procedures (Table 1).

**Table 1.** Tiered system for case designation.

Tier	Designation	Definition	Examples
1	Elective	No likelihood of medical harm from delay	Direct puncture sclerotherapy Angioplasty/stenting for asymptomatic carotid atherosclerotic disease Low risk intracranial aneurysm/vascular malformation Late (>2 year) follow-up angiography for stable treated cerebral aneurysm
2	Urgent	Possible or likely serious medical harm if delayed beyond 30 days	High risk unruptured intracranial aneurysm/vascular malformation Unstable/symptomatic atherosclerotic disease Tumor or infection requiring biopsy to guide management. Tumor requiring preoperative angiography, balloon test occlusion, or embolization for surgical planning/treatment Dural arteriovenous fistula with high-risk cortical venous drainage Pain—minimally invasive spinal procedure^a^
3	Emergent	Serious medical harm possible if any delay	Acute ischemic stroke/ELVO—mechanical thrombectomy Acute intracranial hemorrhage (SAH/ICH/IVH/SDH) requiring diagnostic angiography and/or endovascular treatment of ruptured aneurysm/vascular malformation New neurological deficit/symptoms related to neurovascular pathology requiring diagnostic angiography or endovascular treatment Acute head and neck bleeding Dural venous sinus thrombosis—thrombectomy

a Immobility, reliance on narcotics, depression, or suicidal ideation associated with chronic severe pain represent serious confounding medical/psychological issues that may render a case urgent, especially spinal pain procedures, vertebral augmentation, and minimally invasive spinal decompressive procedures. ELVO: emergent large vessel occlusion; SAH: subarachnoid hemorrhage; ICH: intracerebral hemorrhage; IVH: intraventricular hemorrhage; SDH: subdural hemorrhage.

In addition to categorizing resources utilized and medical risks of delaying procedures, psychosocial and financial pressures facing patients while waiting for their elective procedures must be considered.^31^ This is especially true for those who have lost, or are at risk of losing, their source of income and insurance. Length of time waiting in queue must also be considered due to its psychological toll as well as an issue of fairness.

## Operational logistics

An impartial hospital governing body or committee, consisting of representatives of hospital administration, and representing the diversity of affected procedural service lines may be necessary to adjudicate case priority status. If such a governing body or committee is convened then frequent communications between this body and scheduling physicians should be encouraged to promote transparency and to allow monitoring and reevaluation of cases which may be changing in urgency.

## Specific recommendations for neurointerventional procedures

When the health system and applicable government and regulatory authorities have confirmed conditions and resources are adequate for the performance of elective cases and if necessary after cases have been prioritized, the performance of these procedures may benefit from the following recommendations to safeguard both patients and health care providers.^32–35^ Figure 1 is a printable checklist that embodies the suggestions below. These suggestions are made recognizing the wide variation across the globe with respect to both the burden of the pandemic and resources available to provide care to patients in the pandemic setting:

1. Patients and family members should be educated regarding how undergoing an elective procedure in the pandemic setting affects their risk of becoming infected during the hospital experience and also how their hospital experience may be impacted due to pandemic-specific requirements such as screening, testing, self-isolating, potential re-scheduling, and additional limitations on access to/by visitors. Patients should be given the opportunity to delay the procedure, if desired, to minimize this inconvenience or discomfort.
2. A formal scheme for screening patients should be defined, with screening recommended at time of scheduling the elective procedure, the day before the procedure, and the morning of the procedure. A formal protocol should also be defined for the type and timing of COVID-19 testing for patients who display any risk factors during the screenings, or have a temperature over the limit defined by the institution and any applicable regulatory agencies. Patients undergoing aerosolizing procedures should receive the defined type and timing of COVID-19 testing as appropriate for that region or locale. Any positive results of screening or testing must be communicated between the scheduling team, the procedural team, anesthesia, recovery room, day surgery, and/or post-procedural units as indicated. Consensus should be reached on whether to continue with an elective procedure on a COVID-19 positive or PUI patient, utilizing the hospital's COVID-19 protocols, or whether to reschedule the procedure.
3. The procedural team should confirm post-procedural ICU/hospital bed space availability at the time of scheduling and again the morning of the procedure, and confirmation of both COVID-19 status and availability of anticipated post-procedural bed should be included in the standard pre-procedural time-out.
4. Procedures should be performed as per the institution's standard protocols and COVID-19 policies, with emphasis on enhanced PPE, appropriate donning and doffing of PPE, intubation of COVID-19 positive patients in negative-pressure rooms if available and post-procedural deep cleaning of procedural room and time intervals between cases.

## Conclusion

The COVID-19 pandemic has placed significant strain on health care systems around the globe. Neurointerventional services are vital to patients. Delaying elective procedures may be necessary when acutely ill COVID-19 patients overwhelm resources, but delaying elective procedures is not without risk. Health systems must develop coordinated protocols that define when elective cases can safely be resumed and that promote safe performance of elective neurointerventional procedures during a pandemic.
